# Economically Feasible Mass Production of Egg Yolk Powder Tablets (Chicken IgY) for Global COVID-19 Transmission Prevention

**DOI:** 10.21203/rs.3.rs-5068132/v1

**Published:** 2025-03-19

**Authors:** Michelle Hawkins, Brian Robley, Farhang Alem, Aarthi Narayanan, Philip Larson, Jason Hull, Isabella Hajduk, Michael Wallach

**Affiliations:** Camas Inc.; Camas Inc.; George Mason University; George Mason University; Camas Inc.; Camas Inc.; Centre for Innovative Medical Research; Centre for Innovative Medical Research

**Keywords:** COVID-19, Chicken IgY, Egg yolk powder, Tablets, Passive immunization

## Abstract

Despite the overall positive outcomes in hospitalization and mortality rates from the COVID-19 vaccines, COVID-19 infections remained prevalent around the world highlighting the need for alternative control strategies. Passive immunization with chicken IgY has long served as a feasible countermeasure, which gained further popularity in the research community during the recent pandemic. Here we demonstrate for the first time the scalability of anti-COVID-19 IgY production for effective distribution and potential use in large populations. Over 70,000 chickens were immunized against the SARS-CoV-2 S1 antigen to produce eggs containing anti-S1 IgY. The resulting egg yolk powder was formulated into commercially acceptable tablets for human consumption. QC and stability testing showed that the purified IgY and tablets maintained activity and stability for over a year. The resulting large batch of IgY tablets demonstrated equal immunoreactivity and virus neutralization potential against all leading COVID-19 strains. Our results demonstrate the feasibility of manufacturing egg yolk powder into edible tablets, and that can now be employed to block viral infectivity and transmission against all major COVID-19 strains affordably and effectively manner in both developed and developing countries.

## 1 Introduction

The influx and severity of COVID-19 infections has gone beyond our expectations. The unprecedented number of infections and extremely high rates of transmission have caused significant strain on hospitals and other health care facilities. Despite the use of available PPE (face masks or shields, gloves, disposable gowns), vigilant hand washing and disinfection, as well as vaccines, we have witnessed a large increase in the numbers of COVID-19 cases, particularly with the Omicron strain and its variants. Since the Omicron peak, there has been a sharp reduction in case numbers reaching a plateau, however, this does not reflect the true situation since many, or most COVID-19 cases today never get reported. This not only poses significant risk to the individual infected patient, but also the people around them, both within the healthcare facility (other workers and patients) and outside (general public and family members). Furthermore, new viral strains may suddenly appear that are highly virulent and transmissible. Thus, despite the success of the COVID-19 vaccines in helping to lower morbidity and mortality, there is a need for strategies that can strongly reduce disease transmission and spread.

Over the past few years, we and others have published several articles demonstrating the use of chicken IgY in controlling infection and transmission by COVID-19 [1–4] as well as other viruses such as SARS [5, 6] and Influenza [7]. This was demonstrated by immunizing laying hens with very small quantities of highly purified SARS-CoV-2 S1 glycoprotein [8], leading to a strong antibody response in the sera of the chickens, which then appears in very large quantity of IgY in their egg yolk. Eggs were collected, the yolk separated and the IgY purified. Using this purified antibody, we demonstrated a very high titer against S1 antigen by both ELISA and Western blotting. It was also shown that it can neutralize the virus in vitro, as well as provide in vivo protection against infection in a hamster model [9]. Finally, a Phase 1 clinical trial was carried out demonstrating the safety of IgY as a nasal formulation in humans [1].

Based on the results obtained in animal model studies, it was concluded that chicken IgY raised against the SARS-CoV-2 S1 protein can act as a barrier against infection and last for at least 6–8 hours. We hypothesized that the IgY can be formulated as either a nasal drop or an edible tablet, to be taken every 6–8 hours when encountering potentially infected people. When administered as a tablet, the IgY antibodies will coat the mouth, oropharynx, larynx, and in particular the trachea [13]. As shown in previous studies, when the virus is inhaled, it will be sequestered and neutralized by the IgY, preventing passage of the virus to the lungs where it normally establishes an infection.

In the current research presented here, we demonstrate the ability and feasibility to scale up and produce chicken IgY (in the form of dry egg yolk) in an edible tablet formulation produced on a large scale under food quality production standards. We also show how this can be accomplished and developed new methods for the scale up of immunization, quality control, epitope specificity and stability testing, demonstration of in vitro cross protection, and tablet formulation.

Worldwide, there are approximately 7 billion laying hens [10] which could be used in an emergency anywhere in the world to produce a vast quantity of protective IgY in as little as 30 days from first inoculation of the hens in a very affordable manner. Our working hypothesis is that by producing large numbers of tablets (in the hundreds of millions or billions) commercially at a very cheap cost, they can be used to help control or slow down the transmission and spread of COVID-19 throughout a hospital, school or in the general public. Further, because these tablets contain IgY purified from a natural food product (eggs), these tablets are amenable for use in children, elderly and individuals with sensitivities to eggs. This work is an important step in demonstrating the scalability and use of chicken IgY to control COVID-19 transmission, that can be marketed as a food product for human consumption. Therefore, the objective for this study was to show that these tablets can be manufactured on large scale in an economically feasible manner for the prevention and control of COVID-19 with the intent for the betterment of global health.

## 2 Material and Methods

### 2.1 Antigens

Antigens were sourced from ACROBiosystems Inc. The variant ID, lineage and origin are listed in Table 1 below.

### 2.2 Vaccination protocol for small- and large-scale production

Seven young White Leghorn hens (38 weeks of age) were vaccinated with SARS-CoV-2 S1 antigen. The S1 glycosylated antigen used was produced in HEK cells labeled His and mixed with phosphate buffered saline (PBS) with Emulsigen-D (Phibro Animal Health Products, Teaneck, NJ, USA) following the manufacturer’s instructions at 50% (v/v). Each Hen received 5 μg of antigen diluted in Emulsigen-D twice, two weeks apart, to a total of 10 μg. Hens were vaccinated using a repeater injector and 1 mL was injected into the breast muscle of each bird. The first immunization was administered on one side of the breast and the second on the other side. Eggs were collected at different time points during the immunization schedule as shown in Table 2.

The same vaccination procedure was performed on the large-scale production of IgY in which 73,000 White Leghorn hens (44 weeks of age) were immunized. The sample size was chosen based on previous studies on other antigens where it was found a remarkable level of consistency in the immune responsiveness of vaccinated laying hens against a variety of antigens [11].

Control IgY used in this study was prepared from eggs laid prior to immunization. The small-scale production included IgY pooled from 24 egg yolks, while the large-scale production was prepared from 96 egg yolks. All eggs used were randomly selected.

All laying hens were raised following the National Chicken Council’s recommendations. Farm personnel followed strict environmental policies which included, proper ventilation, indoor protection from predators and weather, and proper lighting which allowed for the ability to monitor disease. Flock health was inspected daily by trained personnel. On-staff veterinarians and poultry nutritionists designed diets to meet the unique physical needs of egg-laying hens which are customized to the age-specific needs of the flock. Feed and water quality were optimized for hen health. All hens were raised without the use of hormones.

No hens or eggs were excluded at any point of the study. No adverse events were observed throughout the duration of the study.

This study is reported in accordance with the ARRIVE guidelines.

### 2.3 IgY extraction and purification from hen egg yolk

Twelve eggs from each vaccination time point (Table 2) were selected at random. The egg yolks were manually separated from the whites, the vitelline membranes pierced, and the egg yolk drained and pooled. The pooled yolk was gently mixed on a stir plate for 10 min at room temperature. 50 mL of homogenized yolks was combined with 150 mL of 4.67% (w/v) polyethylene glycol 8000 (PEG 8000) in PBS. The solution was centrifuged at 10,000 × g for 15 min at 4°C. The resulting supernatant fluid was recovered through cheesecloth and the volume measured. A volume equivalent to one third the volume of the supernatant (~ 50 mL) of 37.5% (w/v) PEG 8000 in PBS was added to the supernatant and mixed gently on a stir plate for 10 min at room temperature. The solution was centrifuged at 10,000 × g for 15 min at 4°C, supernatant was discarded, and the pellet was resuspended with 50 mL PBS. The solution was centrifuged again at 10,000 × g for 15 min at 4°C to remove residual PEG 8000. The supernatant was discarded, and the pellet was resuspended in 50 mL PBS. The purified IgY was filter sterilized through a 0.2 μm PES membrane, aliquoted, and stored at either 4°C or −20°C depending on future use. The purified IgY preparations were analyzed using the A260/280 ratio, and concentrations approximated using the absorbance at 280 nm.

### 2.4 ELISA

Corning Costar 96-well high-binding polystyrene were coated by applying 250 μL of ACROBiosystems recombinant protein (ACROBiosystems, Newark, DE, USA) at 1 μg ml^−1^ in carbonate bi-carbonate (CBC) buffer (pH 9.6) to test wells and CBC buffer only to control wells. Plates were incubated at 37°C for 1 h. Plates were then aspirated, and each well blocked with 390 μL of 1% bovine serum albumin (BSA) for another hour at 37°C. Wells were washed with Tris-buffered saline containing 0.05% (v/v) Tween 20 (WB) and air dried in a 25°C incubator for 1 h. Plates were used immediately upon drying, or vacuum sealed and stored at 4°C. Purified IgY samples to be tested were prepared by initially diluting the antibody 1:600 in BSA and serially diluting 1:2 in BSA. 125 μL of each dilution were transferred to ACROBiosystems recombinant protein-coated microwell plates. Plates were incubated at 25°C for 1 h. The plates were washed three times with 400 μL WB per well. Plates were then conjugated with KPL goat anti-chicken IgY IgG HRP (Horseradish peroxidase; Thermo Fisher Scientific) at a concentration of 100 ng ml^−1^ for 1 h at 25°C. Plates were then washed four times with 400 μL WB per well. The assay was developed in the dark using 100 μL per well of Tetramethylbenzidine (TMB) two component peroxidase substrate system (SeraCare Life Sciences, Inc., Milford, MA, USA) for 10 min. Developing was stopped using 100 μL of 1M sulfuric acid. Absorbance of wells was measured at 450 nm and corrected using control well values.

### 2.5 LDS-PAGE and Western blotting

Western blotting was performed using the iBlot 2 and iBind systems (Thermo Fisher Scientific, Inc., Waltham, MA, USA). Sample preparation for gel electrophoresis was performed following the NuPAGE Bis-Tris Mini Gel protocol (Thermo Fisher Scientific). Every sample was comprised of NuPAGE LDS sample suffer and NuPAGE reducing agent at 1X concentration. The targeted protein amount for each sample was 2 μg/well. Deionized water was added to a final volume of 45 μL. Samples were heated at 100°C for 3 min. Both chambers of a Life Technologies Mini Gel Tank (Thermo Fisher Scientific) were filled with 400 mL of 1X NuPAGE MES running buffer. NuPAGE 4–12% Bis-Tris mini gels were rinsed with 1X running buffer and placed in each chamber of the gel tank. A volume of 20 μL from each sample were loaded into both gels. Electrophoresis was performed at 200 V constant for 30 min. Upon completion, gels were washed in deionized water three times for 10 min each on a platform shaker. One gel was then stained with Imperial Protein Stain (Thermo Fisher Scientific) for final LDS-PAGE analysis, while the duplicate gel was placed on an iBlot 2 PVDF transfer stack.

The transfer was performed on the iBlot 2 system at 20 V for 1 min, 23 V for 4 min, and then 25 V for 2 min per iBlot 2 protocol recommendation. The membrane was then washed in 1X iBind solution and placed in the iBind Western system. anti-SARS-CoV-2 S1 His-tag IgY was used as the primary antibody at a concentration of 15 μg ml^−1^. KPL goat anti-chicken IgY IgG HRP was applied as the secondary antibody at a concentration of 15 μg ml^−1^. After overnight incubation, the membranes were washed for 1 min in deionized water. Detection was performed using Novex HRP chromogenic substrate 3,3’,5,5’-Tetramethylbenzidine (TMB) for 5 minutes on a platform shaker. Membranes were allowed to dry in a dark drawer prior to image capture.

### 2.6 Dot Blotting

2 μL of recombinant protein (S1, RBD, NTD) at a concentration of 0.5 mg ml^−1^ were dotted onto strips of nitrocellulose (Pall Corp., Port Washington, NY, USA). After drying, non-specific sites were blocked in 5% (v/v) BSA. Strips were incubated with anti-S1 IgY or anti-Normal IgY at 25 μg ml^−1^. Strips were washed in 0.05% PBS-Tween 20 (v/v). The strips were incubated in goat anti-chicken IgY IgG HRP-conjugated (Thermo Fischer Scientific) at 1 μg ml^−1^. After washing, the blots were developed with Novex HRP chromogenic TMB (Thermo Fischer Scientific).

### 2.7 RBD binding assay

Two commercial ELISAs manufactured by ACROBiosystems catalog EP-107(WT) and EP-115(B.1.1.529) were used to look at the potential for the IgY to inhibit the RBD binding to the ACE2 receptor. The protocol was provided along with all the reagents by ACROBiosystems. The plate provided was precoated with Human ACE2 protein. Samples and controls were added to the wells along with the HRP labeled SARS-CoV 2 Spike RBD. The plates were incubated for 1 h at 37 °C, developed with TMB, and read at an absorbance of 450nm.

### 2.8 In vitro virus neutralization

The chicken-derived IgY samples labelled Day 0, Day 14, Day 21, and Day 28 were assessed for their ability to neutralize SARS-CoV-2 virus using a PRNA assay. Briefly, the antibodies were diluted 1:10, 1:20,1:40, and 1:80 in culture medium and incubated with 200 plaque forming units (PFU) of the Washington Strain of SARS-CoV-2 (USA-WA1/2020). SARS-CoV-2 was diluted in supplemented (Dulbecco’s Modified Eagle’s Medium) DMEM to the appropriate concentration to achieve 200 PFU. The virus was then added to antibody samples and allowed to incubate for 1 h at 37 °C and 5% CO_2_. After incubation, a viral plaque assay was conducted to quantify viral titers using 12-well plates previously seeded with Vero cells (ATCC CCL-81) at a density of 2 × 10^5^ cells per well. Media was aspirated from plates and virus-antibody samples were transferred to wells, one sample per well. Plates were inoculated for 1 h at 37 °C and 5% CO_2_. After infection, a 1:1 overlay consisting of 0.6% agarose and 2X Eagle’s Minimum Essential Medium without phenol red (Quality Biological, 115-073- 101), supplemented with 10% fetal bovine serum (FBS) (Gibco, 10,437,028), non-essential amino acids (Gibco, 11140–050), 1 mM sodium pyruvate (Corning, 25-000-Cl), 2 mM L-glutamine, 1% penicillin-streptomycin (P/S) was added to each well. Plates were incubated at 37°C for 48 h. Cells were fixed with 10% formaldehyde for 1 h at room temperature. Formaldehyde was aspirated and the agarose overlay was removed. Cells were stained with crystal violet (1% w/v in a 20% ethanol solution). The viral titer of SARS-CoV-2 was determined by counting the number of plaques.

### 2.9 Tablet formulation

Seattle Gourmet Foods (Kent, WA, USA), a commercial candy manufacturer, was contracted for small pilot runs and a scale up to a production run. The edible tablets produced contained Dipack (sugar and maltodextrin; 49.5%), dextrose (26.5%), pasteurized egg yolk powder (15%), natural flavor (3.5%), and citric acid, malic acid, stearic acid, and magnesium stearate (≤ 2% each). The powders were mixed and made into tablets using a tablet press.

### 2.10 Statistical analysis

The statistical analyses were analyzed using the GraphPad Prism 8.0.1 software (GraphPad Software, Boston, Massachusetts USA). Statistical significance was performed using the T-test (2-tailed), where *p* < 0.05 was considered to be statistically different.

## 3 Results

### 3.1 Immunoreactivity of IgY against SARS-CoV-2 S1 protein tested on a small scale.

**Figure 1:**
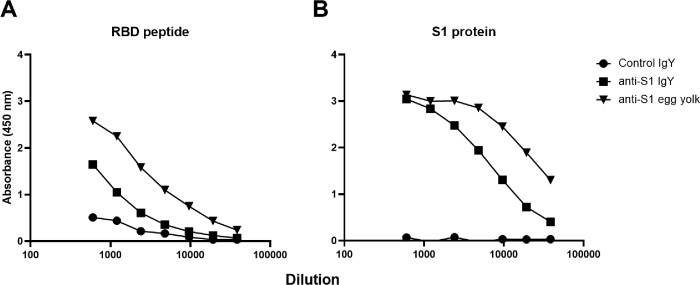
Immunoreactivity of IgY raised against S1 protein. Immunoreactivity of IgY via enzyme-linked immunosorbent assay (ELISA), in either purified or non-purified (egg yolk) form was tested against (A) the Receptor-Binding Domain (RBD) epitope or (B) whole construct of the SARS-CoV-2 S1 protein.

The SARS-CoV-2 S1 protein or RBD peptide reactivity of both purified IgY and egg yolk was examined in eggs collected from 7 hens at two weeks post second immunization (D21) with Fc tagged S1 glycoprotein using ELISA. The ELISA assay (Figure 1) showed strong positive binding of both egg yolk-IgY and purified IgY to the S1 protein demonstrating that the S1 protein is highly immunogenic in hens. These antibodies also reacted very strongly with the RBD peptide by ELISA. Finally, there was only a small decrease in binding affinity of the purified anti-S1 IgY compared to that of the IgY in whole egg yolk.

### 3.2 Immunoreactivity of purified IgY from individual eggs

**Figure 2:**
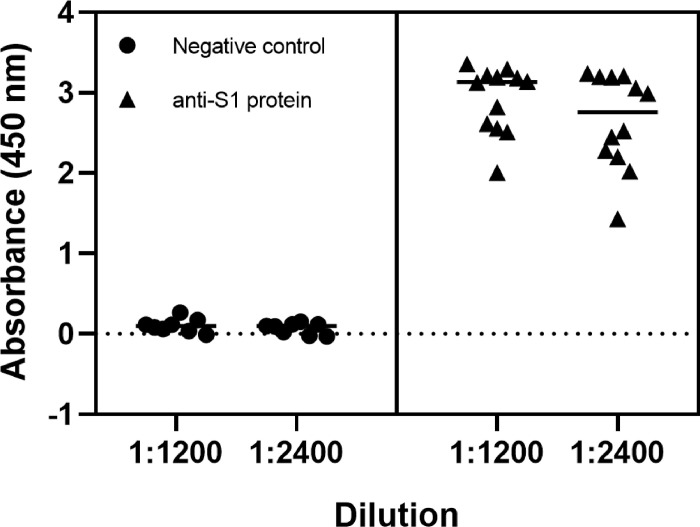
Immunoreactivity of purified IgY from individual eggs. IgY purified from individual eggs from hens not immunized (left; negative control) or immunized (right) against SARS-CoV-2 S1 protein. Immunoreactivity of IgY was tested at two dilution (antigen: IgY) ratios: 1:1200 and 1:2400. Statistical difference determined between the negative control and test samples (*p* < 0.0001).

Individual eggs may demonstrate different IgY content leading to variability during production and manufacturing. Therefore, to determine the extent of variability of the IgY titer in individual hens, 12 eggs from different immunized hens were selected at random and their IgY isolated and tested by ELISA. This was compared to 8 eggs collected at random from non-immunized hens. As shown in Figure 2, we found that the IgY titer was high in all the eggs from immunized hens with relatively low variability. In contrast, the negative controls (IgY from non-immunized hens), only gave consistent background levels of reactivity.

### 3.3 Immunoreactivity of purified IgY over time

**Figure 3:**
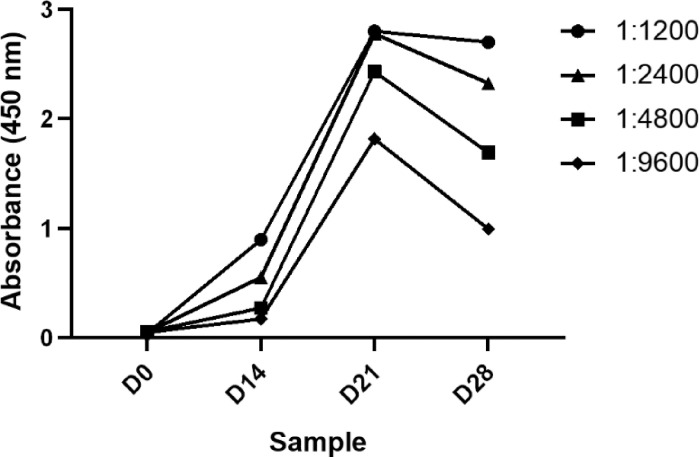
Immunoreactivity of purified IgY at different time points. Reactivity of IgY purified from eggs collected at weekly intervals was tested via ELISA. Immunoreactivity of IgY was tested at different antigen:IgY dilution ratios.

To determine the rate at which anti-S1 antibody activity appears in the egg yolk, an ELISA was performed on purified egg yolk IgY taken at each stage of egg collection. As shown in Figure 3, no increase in IgY titer is seen at the first egg collection after the first immunization (D0). However, a significant increase in IgY titer is seen at the third egg collection (D21). The high IgY titer is maintained for the remaining samples collection over a period of 3 weeks and further work showed that the titer remains high for 3–4 months (data not shown).

### 3.4 Western Blot Analysis of IgY

**Figure 4:**
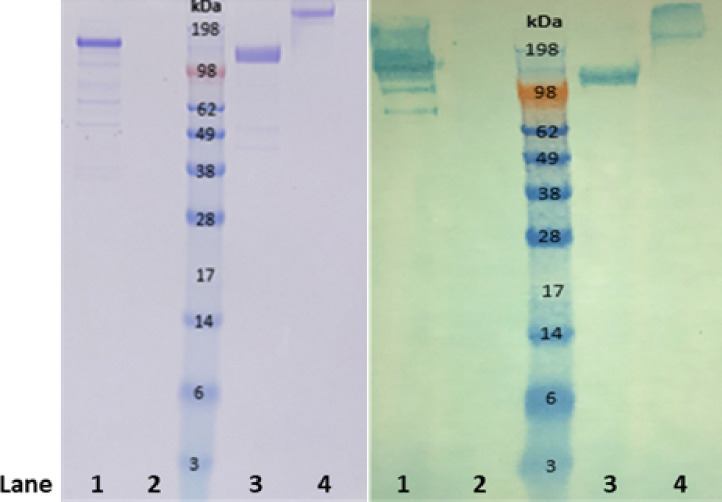
Protein analysis of antigen specificity. Left: SDS-PAGE analysis of α-ACROBiosystems SARS-CoV-2 S1 IgY and ACROBiosystems SARS-CoV-2 S1 Recombinants – reduced and non-reduced. Right: Western Blot Analysis of α-ACROBiosystems SARS-CoV-2 S1 IgY and ACROBiosystems SARS-CoV-2 S1 Recombinants – reduced and non-reduced. Lanes are identified as follows: (1) α-ACROBiosystems SARS-CoV-2 S1 IgY (Lot#061820BR) positive control; (2) 1X LDS sample buffer negative control (Lot#2165459) (3) ACROBiosystems SARS-CoV-2 S1 recombinant (Lot#3594b-203ZF1-RC), reduced; (4) ACROBiosystems SARS-CoV-2 S1 recombinant (Lot#3594b-203ZF1-RC), non-reduced. Each lane is loaded with 2 ug targeted amount. Original full-length images can be seen in Figure S1.

LDS gel and Western blot analysis was conducted to confirm both the purity of the IgY and its reactivity against the S1 antigen (reduced and non-reduced; ACROBiosystems). HRP conjugated goat anti-chicken IgY reacted strongly with a 180 kDa protein band of IgY under non-reducing conditions (Figure 4, lane 1). The IgY reacted strongly with both reduced and non-reduced forms of the SARS-CoV-2 S1 antigen, suggesting the IgY antigen recognition site is not affected in reduced conditions.

### 3.5 Virus Neutralization Study

**Figure 5:**
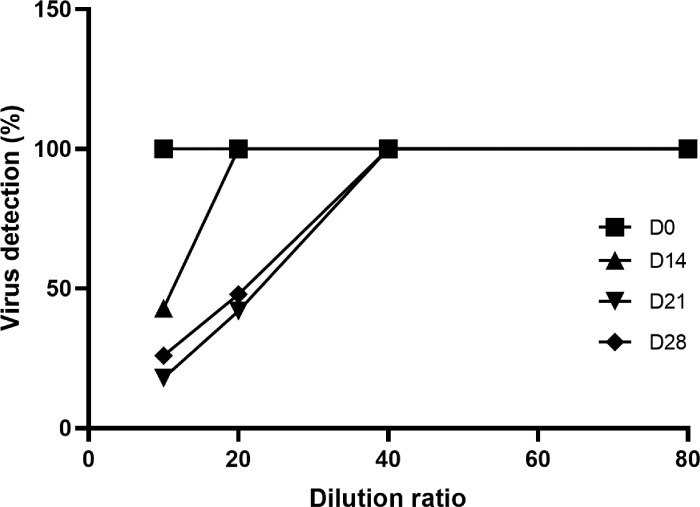
In vitro virus neutralization on the small-scale IgY production. Plaque formation was quantified in Vero cell cultures infected with the SARS-CoV-2 alpha variant and co-cultured with different dilutions of anti-SARS-CoV-2 IgY collected at different time points post immunization.

IgY from hens immunized with the S1 antigen were tested using an in vitro plaque assay for virus neutralization. This assay was completed under blinded conditions. The Alpha variant of SARS-CoV-2 was used to infect Vero cells in culture. There was a significant reduction in plaque forming units reaching a maximum at 3 weeks post second immunization (D28; Figure 5). Interestingly, there was a significant reduction seen on D14 with a titer of 1:10 reaching a maximum titer of 1:20.

### 3.6 Upscaling of IgY production

**Figure 6:**
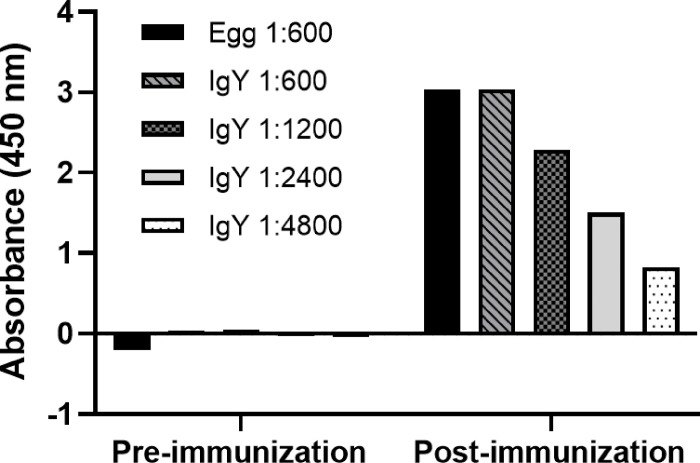
IgY titer post-immunization in large-scale production. Reactivity of purified (IgY) or non-purified (Egg) IgY from eggs was tested via ELISA. Immunoreactivity of IgY was tested at different antigen:IgY dilution ratios.

We next carried out large-scale production of the SARS-CoV-2 S1-specific IgY by vaccinating 73,000 hens with 5 μg of purified his tagged S1 glycoprotein. An ELISA was performed to examine the IgY titer in both the purified IgY fractions and in the egg yolks sampled post immunization (Figure 6). Consistent with the small-scale experiments, a significant increase in the SARS-CoV-2 S1 IgY in both purified IgY samples and egg samples was seen post immunization with the SARS-CoV-2 S1 protein antigen.

Following confirmation of the high titer of IgY in the egg yolks of the immunized hens, eggs were collected and sent to a commercial egg processing facility where the egg yolks were separated, pasteurized, and spray dried. After 12 weeks of collection and pooling into batches we produced a total of 63,000 kg of high titer, spray dried egg yolk powder.

To ensure that the spray dried egg yolk was free of contaminating pathogens, a quality control and batch safety test was carried out. The dried egg yolk was tested for the presence of common contaminant pathogens. No significant levels of contaminants were detected in the spray dried egg yolk which were therefore approved for food safety (Table 3).

### 3.7 Production of tablets does not affect IgY titer.

**Figure 7:**
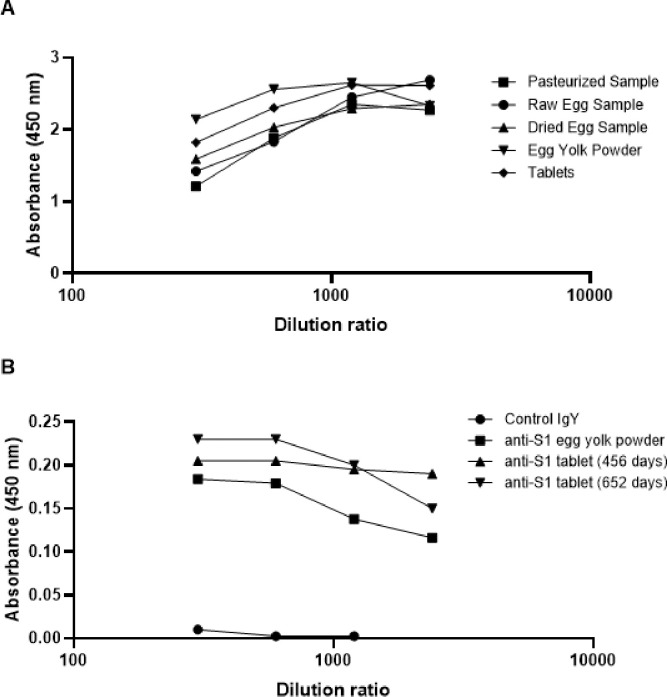
Immunoreactivity of IgY throughout the (A) production and (B) storage process. Tablets were stored in a dark room at room temperature from the date of manufacture to the date of testing. Sample concentrations were normalized before plotting. Immunoreactivity of IgY was tested at different antigen:IgY dilution ratios.

Tablets were produced on a large scale at the approved facility of Seattle Gourmet Foods, Washington, USA. The final prototype product contained 120 mg of egg yolk powder in a tablet weighing 800 mg. To ensure production and manufacturing of dried egg yolk into the final tablet form does not affect the quality of the IgY, antibody titer was established using ELISA at the different stages of production. These stages included the collection of large quantities of yolk from eggs of immunized hens, pasteurization of the egg yolk, production of egg yolk powder by spray drying and formulation into a tablet. It was found that the whole process from egg to the production of the tablets was found to not significantly impact the titer of the IgY in the product (Figure 7a).

Given that the intended tablets would be used over a period of time, we wanted to ensure that the IgY titer maintained stability throughout its expected 1-year shelf life. Tablets were stored at room temperature long-term (nearly 2 years (652 days)) were tested by ELISA (Figure 7b). It was found that IgY titer levels in the tablets remained stable throughout the entire storage period as compared to the positive egg-yolk powder control.

### 3.8 Chicken IgY antibody titer against SARS-CoV-2 variants

During the time of the scale up production of the chicken IgY, several variants of SARS-CoV-2 arose in various countries around the world. Available vaccines against SARS-CoV-2 have shown variable efficacies against these new variants. It therefore raised the question of the reactivity of Chicken IgY raised against the alpha strain S1 glycoprotein, against these new SARS-CoV-2 variants.

**Figure 8:**
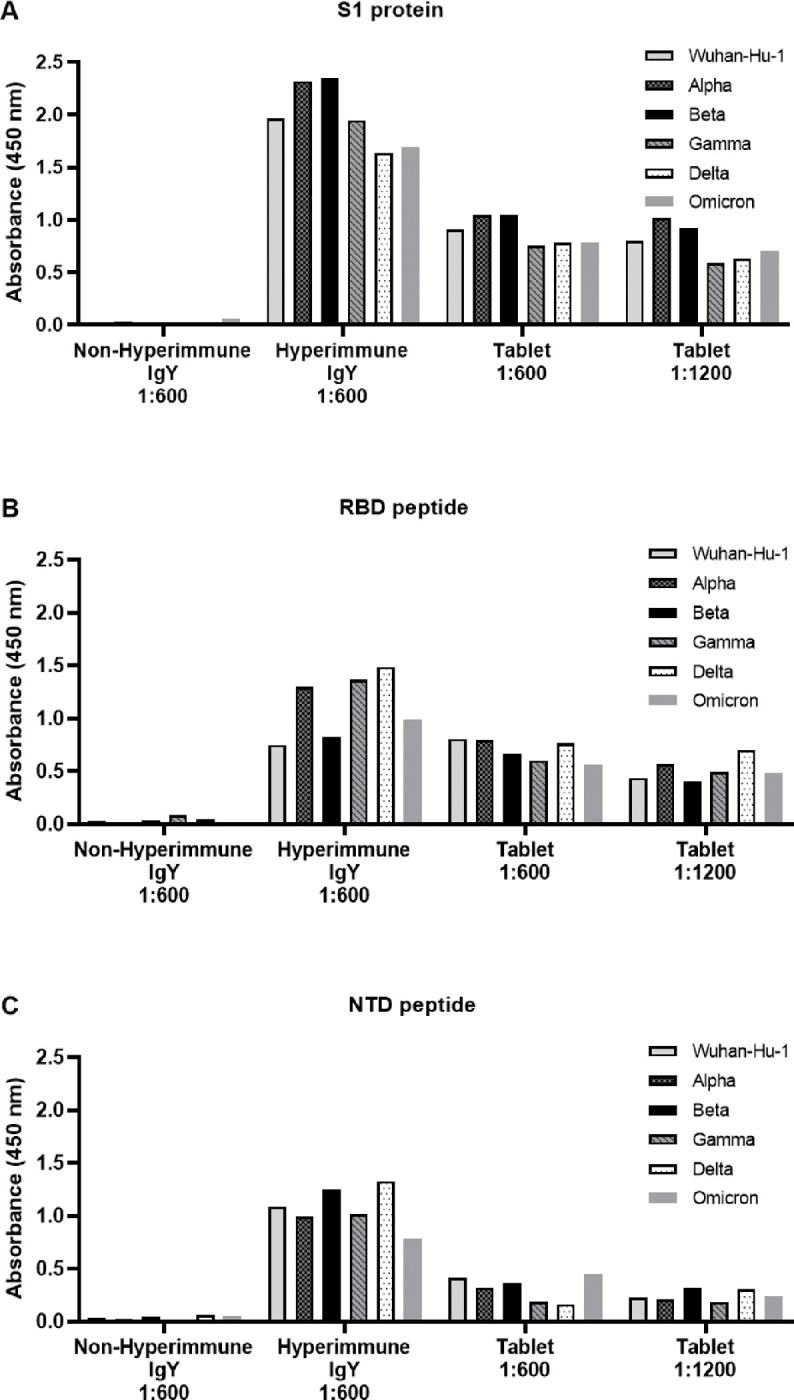
Immunoreactivity of IgY to the different forms or fractions of some of the leading COVID-19 variants: (A) S1 Protein, (B) RBD peptide and (C) NTD peptide. Immunoreactivity of IgY was tested as a purified form (Anti-S1 IgY).

The SARS-CoV-2 S1 spike protein is composed of two key epitopes: a ribosomal binding domain (RBD) and an N-terminal domain (NTD). The IgY has been raised against the whole S1 protein, and we wished to test its binding affinity to the S1 protein as well as these two specific regions. An ELISA was performed to determine the cross reactivity of anti-Wuhan strain IgY against the S1 protein, RBD and NTD from all the leading SARS-CoV-2 variants. As can be seen in Figure 8A, it was found that antibodies against the original Wuhan S1 protein reacted strongly with the S1 protein from all the variants tested with OD values between 1.5–2.5 absorbance units at a dilution of 1:600. In contrast, control, non-immune IgY had no significant reactivity with any of the proteins/peptides tested.

The results also showed that there was very strong cross reactivity of the anti-S1 IgY against all RBDs and NTDs tested (Figure 8B and 8C, respectively) and as expected the OD values were somewhat lower (between 1–1.5 absorbance units) than that found against the whole S1 protein. Both purified IgY and IgY extracted from the tablets fully cross reacted against the S1s, RBDs and NTDs. Finally, it was found that the reactivity with the RBD peptide was significantly stronger than that seen with the NTD peptide particularly when using tablet IgY.

### 3.9 Cross reactivity of Chicken IgY against SARS-CoV-2 variants by Western blotting and dot blotting

**Figure 9:**
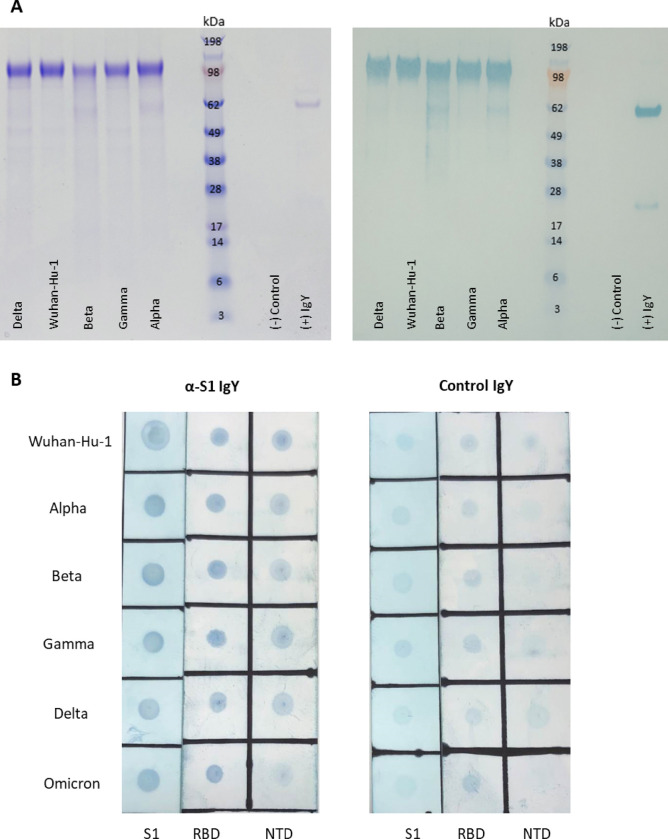
(A) Coomassie stained LDS-PAGE (left) and Western Blot (right) of recombinant whole S1 variants. Each lane is loaded with 2 ug targeted amount. All samples were reduced. Western blot was performed using anti-S1 IgY as the primary antibody. (B) Immunoblot of RBD and NTD variants against anti-S1 IgY (left) and control IgY (right). Original full-length images can be seen in Figure S1.

**Figure 10:**
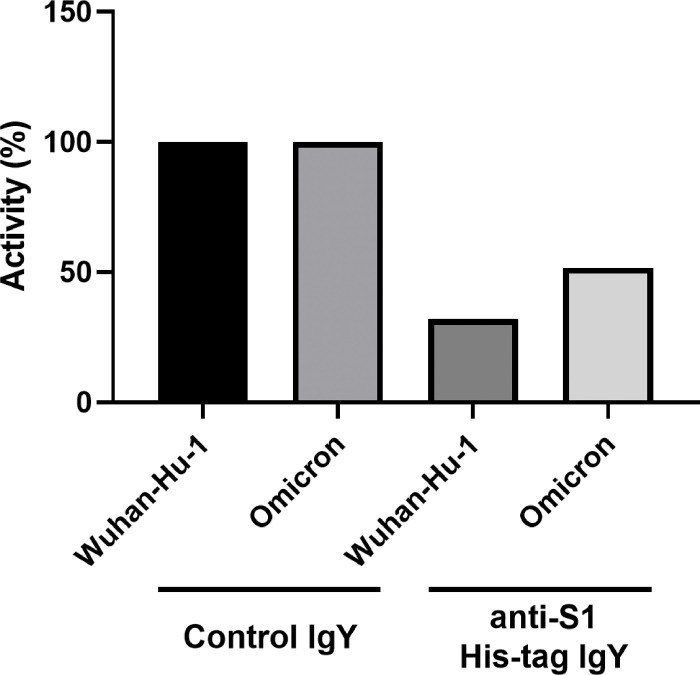
ACROBiosystems SARS-CoV-2 spike RBD/ACE2 binding inhibition assay. Percent of activity remaining from inhibition of control IgY and anti-S1 IgY against either the Wuhan-Hu-1 or Omicron variant. Statistical difference determined between the IgY control and anti-S1 samples (*p* < 0.05).

Western blot and dot blot analysis were used to examine the specificity of IgY to the whole S1 as well as to the RBD and NTD epitopes of the antigen, respectively (Figure 9 and 10). We found that there was equal reactivity against the S1 glycoproteins in all the strains tested by Western blotting as was seen by ELISA (Figure 9a). To reduce any possible effect of incomplete transfer during blotting, we also carried out a dot blot analysis comparing S1, RBD and NTD glycopeptides from all the major variant strains. Once again as seen by ELISA there was little or no difference in intensity between the variants (Figure 9b). These results taken together show that the IgY recognized the major epitopes in S1 equally in all variants.

### 3.10 In vitro RBD/ACE2 binding inhibition assay

An RBD/ACE2 binding inhibition assay was performed to test the ability of the anti-S1 IgY to block the interaction between these two key receptor proteins. As can be seen in Figure 10, there was a strong reduction in RBD binding to ACE2 in both the original Wuhan Hu-1 strain and the Omicron variant strain. This further supports the concept of using S1 to immunize hens giving rise to IgY that can inhibit virus binding to its host receptor.

### 3.11 In vitro Virus Neutralization test using IgY.

**Figure 11:**
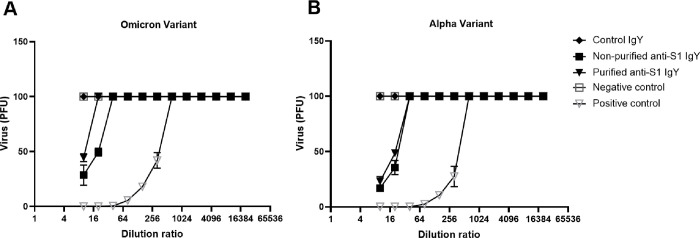
In vitro virus neutralization on the large-scale IgY production. Plaque formation was quantified in Vero cell cultures infected with the SARS-CoV-2 variants (A) Omicron (SARS-CoV-2 Isolate hCoV-19/USA/MD-HP20874/2021 (Lineage B.1.1.529; NR-56461)) and (B) Alpha (SARS-Related Coronavirus 2 isolate Italy-INMI1 (NR-52284)), and co-cultured with different dilutions of anti-SARS-CoV-2 IgY collected.

An IgY sample purified from the large-scale egg yolk powder production batch provided good in vitro neutralization against both the Omicron strain (NR-56461 from BEI resources, Manassas, Virginia), and the Italian Alpha strain of SARS-CoV-2 (NR-52284), reaching a titer of 1:20 at a concentration of 9.3 mg ml^−1^ (Figure 11) in comparison to control IgY used at a concentration of 8.7 mg ml^−1^ that showed no significant neutralization of the virus.

## 4 Discussion

In this paper it was demonstrated that commercial hens raised under farm conditions and vaccinated on a large scale (73,000 hens), mount a very strong immune response when immunized with very small quantities of SARS-CoV-2 S1 glycoprotein produced in HEK cells (5 μg per immunization given twice intramuscularly & spaced two weeks apart). Based on the ELISA results, the titer of the egg yolk antibody is very high and long lasting (up to 4 months post second vaccination). In addition, 100% of the hens responded to vaccination with all the eggs collected showing a very large quantity of anti-S1 IgY in the egg yolks, which based on previous studies has been shown to contain 100–150 mg specific IgY per yolk [7].

Studies carried out using ELISA, Western and Dot blotting, demonstrated that the IgY produced against S1 from the Wuhan strain cross-reacted very strongly with all 6 of the major SARS-CoV-2 strains that have appeared globally (Wuhan original strain, Alpha, Beta, Gamma, Delta, and Omicron). The reason for this is associated with the ability of Chicken IgY to cross react with different strains of viruses as was found with influenza [7] and other viruses of human and veterinary importance.

Our epitope mapping results using the SARS-CoV-2 S1 glycoproteins and RBD and NTD peptides in ELISA, showed the excellent cross reactivity between all the variant derived molecules/epitopes. The ELISA results showed that the reactivity against S1 was the strongest followed by the RBD and NTD peptides as expected. Research [12, 13] into the RBD has highlighted its important role in establishing disease via its binding to the ACE2 receptor. In addition, recent studies [14, 15] have revealed a potential role for the NTD during infection and have suggested a combined RBD and NTD approach of virus neutralization may be a better way forward in disease treatment. The finding that the IgY produced against S1 reacted well with all variant RBD and NTD peptides strengthens the case for its potential use throughout the pandemic, regardless of which variant arises in the population.

In a recent study carried out by Li et al. [16], those researchers mapped 20 peptide epitopes with relatively strong signals in the S-protein using IgY produced against the extracellular domain of the S-protein. Interestingly, those researchers found that 17 of them are highly conserved between SARS-CoV-2 variants with no mutations seen in the amino acid sequence. In addition, they found that the IgY used in mapping the peptide epitopes had a 98% neutralization capability. These results help explain the strong cross reactivity we observed between the SARS-CoV-2 variants and further support the concept of using IgY as a means of providing passive protection against the disease.

Virus neutralization testing using the alpha strain demonstrated the ability of IgY to block virus infection based on a plaque assay in Vero cells. It also shows that IgY made against the Wuhan strain can cross protect against infection by the Washington and even Omicron strains. These results are supported by the RBD/ACE2 binding inhibition assay showing inhibition of binding to the RBD. Together with previous results seen using IgY to protect hamsters against COVID-19 infection, they demonstrate the potential of this approach to control COVID-19 transmission.

A study carried out using mice and hamsters to test IgY raised against the RBD followed by challenge infections with SARS-CoV-2 RBD, demonstrated the safety and protective effect of this approach [9]. Hamsters treated with IgY intranasally before or after infection with SARS-CoV-2 showed a clear inhibition of lung pathology. In addition, the IgY was shown to remain in the upper airways for several hours. Similar results were demonstrated by Wongso, et al., who found that IgY administered intranasally in mice accumulated in the trachea [17]. In our approach using the tablet formulation we would expect the IgY to also remain in the upper airways/trachea for at least 6–8 hours. Further work is required to directly demonstrate this persistence of the antibody in humans.

Quality control of the large batch of IgY produced from 73,000 immunized hens showed that it reacted well by ELISA, Western and in vitro neutralization against all variant strains. The final tablet formulation showed that the IgY was non-contaminated based on microbiology and had a good neutralization titer. The final tablet formulation also showed good stability of the egg yolk powder IgY. Previous results have shown that IgY is very stable when produced as egg yolk powder [18]. However, we needed to show that this is also the case when formulated into a tablet. Based on our ELISA results comparing IgY in egg yolk powder (positive control) to that in the tablet, we found that there was no decrease in titer over a period of nearly 2 years of storage. These results demonstrate the stability and storability of IgY in a tablet where we expect to receive approval for use after at least 1 year of production. These results demonstrate that IgY can be stockpiled for use against viruses that undergo mutation and variation due to their cross protective capability.

Our results show that IgY can be rapidly manufactured and formulated into a tablet with a cost of production of around 1–2 cents per tablet. It can be used prophylactically to prevent the transmission of SARS-CoV-2. In addition, IgY could potentially be used therapeutically to treat patients, however, it would need to be formulated for intravenous or intraperitoneal injection requiring approval by regulatory authorities.

## 5 Conclusion

In a pandemic characterized by a threat with a high reproduction rate like SARS-CoV-2, prompt implementation of solutions to curb transmission within a population may offer more significant benefits than later measures focused on individual protection. Vaccinating laying hens is vastly quicker and safer compared to the time-consuming processes involved in developing, testing, manufacturing, and distributing novel human vaccines. With IgY, effective antibodies can be produced worldwide immediately upon the identification and generation of viral antigens, requiring only 30 days from the start of immunization with those antigens. This approach has been shown to be plausible against a variety of antigens (reviewed by Lee et al. [11]), highlighting the broad applicability of this cost-effective approach for future pandemics. Thus, transmission can be greatly reduced, buying time for human vaccines to be tested for safety and efficacy and to be distributed. Additionally, sites of egg production and consumption tend to be in close proximity allowing for the leverage of existing infrastructure to produce and process food grade eggs in a geographically efficient manner to quickly respond and deliver protective IgY to help mitigate transmission in the early days of emerging pandemic threats.

## Figures and Tables

**Table 1:** ACROBiosystem recombinant proteins used in this study.

Variant ID	Origin	Lineage	Epitope
Wuhan-Hu-1	China	WT	RBD
			S1
Alpha	United Kingdom	B.1.1.7	S1
Beta	South Africa	B.1.351	RBD
			S1
Gamma	Brazil	P.1	NTD
			RBD
			S1
Delta	India	B.1.617.2	RBD (L452R, T478K)
		B.1.617	RBD (L452R, E484Q)
			NTD
			S1
Omicron	South Africa	B.1.1.529	S1

Abbreviations: wild-type (WT), receptor-binding domain (RBD), N-terminal domain (NTD).

**Table 2:** Timing of egg collection in relation to immunization

Date	Egg Collection Details	Sample Abbreviation
07/09/2020	First round of vaccination	-
14/09/2020	First egg collection	D0
21/09/2020	Second round of vaccination	-
28/09/2020	Second egg collection	D14
05/10/2020	Third egg collection	D21
12/10/2020	Fourth egg collection	D28

**Table 3:** Microbial contamination of IgY samples. Four sample pools of IgY were generated through the pooling of three randomly selected IgY samples in each pool. Colony forming units (CFU) were quantified in each pool for the listed contaminants.

Contaminant	Sample Pool				Quality control specification
	1	2	3	4	
Coliform	< 10	< 10	< 10	< 10	< 10
*E. coli*	< 10	< 10	< 10	< 10	< 10
*Staphylococcus sp.*	< 10	< 10	< 10	< 10	< 10
Yeast/mold	< 10	< 10	< 10	< 10	< 10
*Salmonella sp.*	NEG	NEG	NEG	NEG	NEG
*Listeria monocytogenes*	NEG	NEG	NEG	NEG	NEG
Standard Plate Count	< 100	100	100	< 100	< 5000

## Data Availability

The datasets during and/or analysed during the current study available from the corresponding author on reasonable request.

## Abbreviation

ACE2: angiotensin converting enzyme 2
ATCC: American type culture collection
BSA: bovine serum albumin
CBC: carbonate bi-carbonate
COVID-19: coronavirus disease 2019
DMEM: Dulbecco’s Modified Eagle’s Medium
ELISA: enzyme-linked immunosorbent assay
FBS: fetal bovine serum
HEK: human embryonic kidney
HRP: horseradish peroxidase
ID: identification
IgA: Immunoglobulin Y
IgG: Immunoglobulin G
LDS: lithium dodecyl sulfate
MES: 2-Morpholinoethanesulphonic acid
NTD: N-terminal domain
OD: optical density
PAGE: polyacrylamide gel electrophoresis
PBS: phosphate buffered saline
PEG: polyethylene glycol
PES: polyethersulfone
PFU: plaque forming units
PPE: personal protective equipment
PVDF: polyvinylidene difluoride
QC: quality control
RBD: ribosomal binding domain
SARS: severe acute respiratory syndrome
SDS: sodium dodecyl-sulfate
TMB: tetramethylbenzidine
WT: wild-type
